# Sigmoid volvulus; a rare complicated presentation of Hirschsprung's disease: A case report^☆^

**DOI:** 10.1016/j.ijscr.2021.106608

**Published:** 2021-11-18

**Authors:** Mostafa Zain, Mohamed Abouheba

**Affiliations:** Faculty of medicine, Alexandria university, Egypy

**Keywords:** Sigmoid volvulus, Hirschsprung, Megacolon, Case report

## Abstract

**Introduction:**

Sigmoid volvulus (SV) is a rare complication of Hirschsprung's disease (HD) with only 31 cases have been reported in the English literature. Although its diagnosis is challenging, unrecognized SV is a life-threatening condition requiring early recognition to decrease morbidity and mortality.

**Presentation of case:**

A 14-year-old male presented to our emergency department with massive abdominal distention. Plain erect abdominal X-ray showed massive colonic distention with multiple fluid levels. Colonoscopy failed to pass beyond 15 cm after entering dilated sigmoid loop. Open surgical exploration was done through a lower midline incision and revealed SV with massive distention of the entire colon. After detorsion, we found a markedly dilated sigmoid colon with an evident discrepancy at the lower sigmoid. Due to massive colonic dilatation, the decision was made for terminal ileostomy. Histopathological examination of biopsy from the narrow segment demonstrated aganglionosis and hypertrophic submucosal neural fibers confirming the diagnosis of HD.

**Discussion:**

SV is a rare serious complication of HD. Unrecognized SV is a serious life-threatening condition, so it should be considered in children with acute or recurrent abdominal pain, distension, constipation and vomiting as early recognition and management of volvulus is essential to decrease morbidity.

**Conclusion:**

The presented case highlights the possibility of SV as a rare complication of HD should be considered especially in children with a history of chronic constipation and abdominal distension who present with acute colonic distension and failure to decompress despite rectal washes.

## Highlights

• Sigmoid volvulus is a rare complication of Hirschsprung’s disease with a reported prevalence of 0.66%.
• Prompt diagnosis of the acute of volvulus decreases morbidity and increases survival.
• Sigmoid volvulus should be considered in children presenting with acute intestinal obstruction.
• In children with sigmoid volvulus, Hirschsprung’s disease should be suspected, and proper evaluation is required.

## 1 Introduction

Hirschsprung's disease (HD) result from absence of ganglion cells from the submucosal and myenteric plexus in the intestinal wall, which starts at the internal anal sphincter and extend upward to varying distances [1].

It has a wide clinical spectrum ranging from minimal symptoms to complete intestinal obstruction. In later childhood, HD usually present as chronic constipation may be complicated by enterocolitis, and usually associated with growth retardation [2].

Sigmoid volvulus (SV) is a rare complication of HD with a reported prevalence of 0.66% [3]. Conversely, in children presenting with SV, 18% also had HD [3], [4].

To the best of our knowledge, there are only 31 cases have been reported in the English literature [5]. The work has been reported in line with the SCARE criteria [6].

## 2 Case report

A 14-year-old male presented to our emergency department with respiratory distress due to massive abdominal distention. He reported non- bilious vomiting and decreased oral intake for the past 3 days. His last bowel movement was 5 days prior to the admission. Parents reported that the child had chronic constipation since birth with bowel movement every 2–3 days. He was not on any regular medications but occasionally, he takes oral laxatives, glycerin suppositories and sometimes he may require enema for colonic evacuation. The parents cannot remember when the child passed his first meconium. His growth and development are average for his age. There was no family history of similar illnesses.

**Fig. 1:**
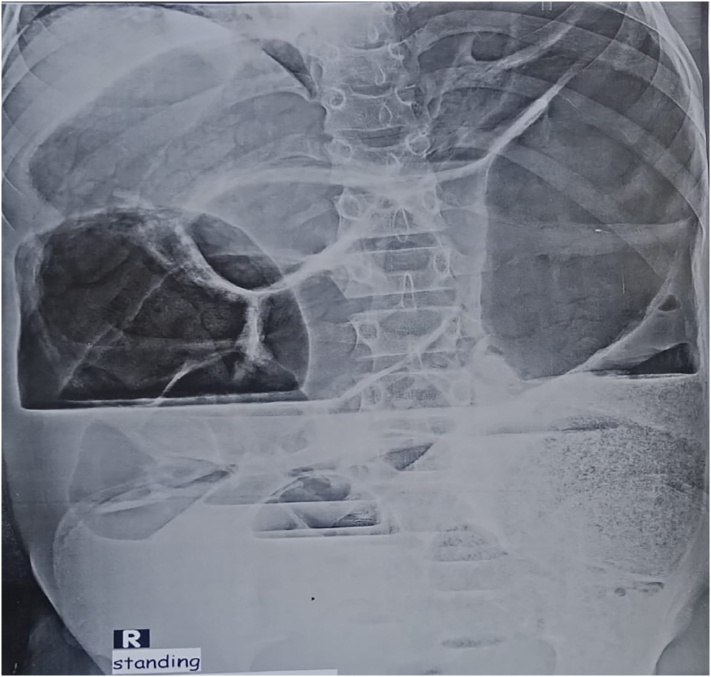
Plain erect abdominal X-ray: massive colonic distention with multiple fluid levels and ground-glass appearance suggesting fecal impaction at the distal part of the sigmoid colon.

On general examination, vital signs were all in normal range except for tachycardia. All anthropometric measurements were in the normal range. Clinical examination of the abdomen showed massive universal distention, tympanic on percussion with diminished bowel sounds. Digital rectal examination revealed empty rectum. Plain erect abdominal X-ray showed massive colonic distention occupying most of the abdomen with multiple fluid levels and ground-glass appearance suggesting fecal impaction at the distal part of the sigmoid colon (Fig. 1).

**Fig. 2:**
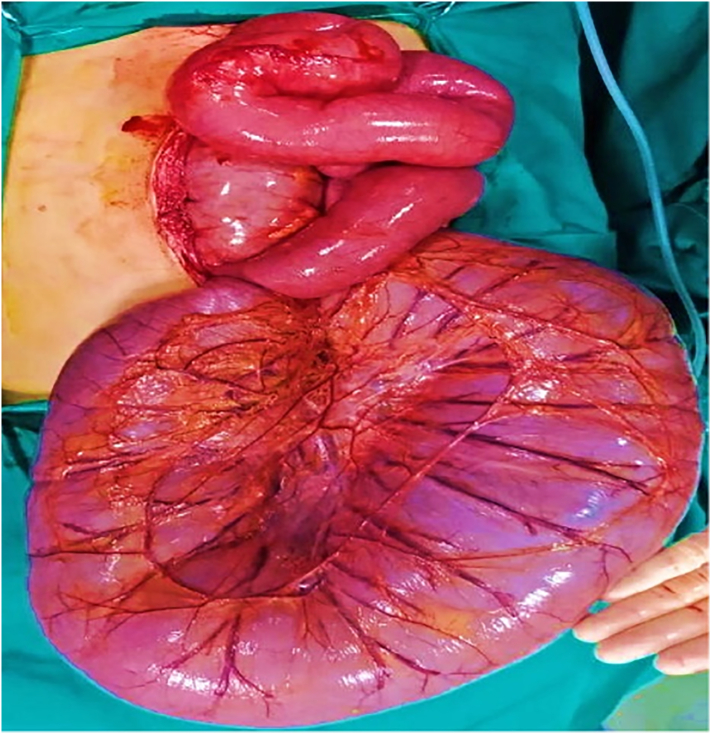
Intraoperative photo showing massively distended sigmoid colon twisted around its base.

After stabilization of the general condition with intravenous fluids, intravenous antibiotics, and correction of hypokalemia, the patient underwent a trial of surgical disimpaction with rectal tube for deflation, which failed to evacuate the colon or decompress the abdomen. Colonoscopy failed to pass beyond 15 cm after entering dilated sigmoid loop. So, the decision was made for surgical exploration. The time between presentation and surgery was about 6 h. The patient was not eligible for laparoscopy due to the massive abdominal distention causing respiratory distress. Open surgical exploration was done by the authors through a lower midline incision and revealed sigmoid volvulus 270 degree clockwise with massive distention of the entire colon (Fig. 2).

**Fig. 3:**
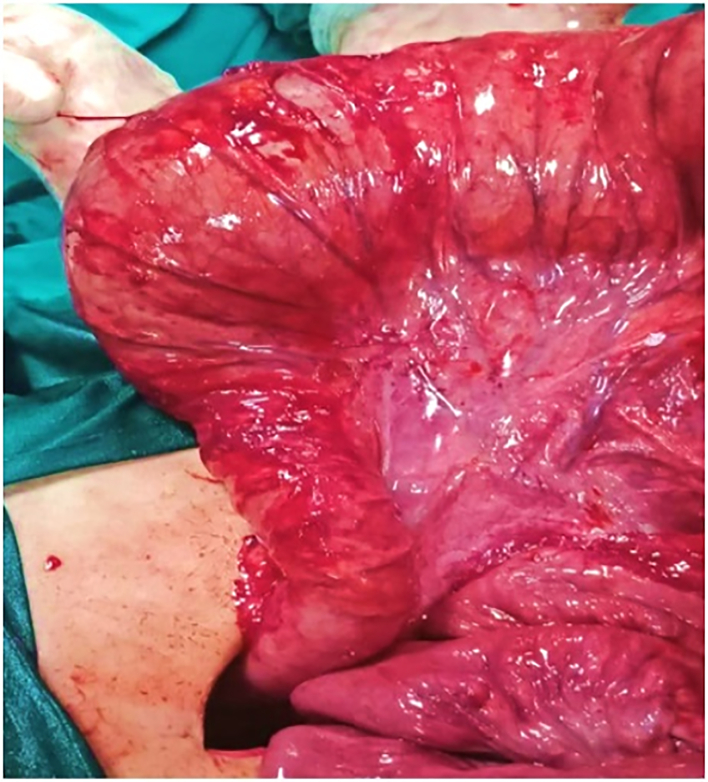
Intraoperative photo showing evident classical funnel shaped discrepancy at the lower sigmoid colon suggestive of HD.

After detorsion of the volvulus, we found a markedly dilated sigmoid colon (length ∼50 cm and diameter ∼15 cm) with an evident classical discrepancy at the lower sigmoid suggestive of HD (Fig. 3).

Due to massive colonic dilatation and ectasia, the decision was made for terminal ileostomy. Evacuation of the colon was made from the ileostomy and by milking toward the anus. Full thickness biopsies were taken from the distal narrow segment, the dilated proximal segment and from the ileostomy. The site of the discrepancy was marked with two 3/0 sutures to help in the future pull through operation. The patient started oral feeding on the 2nd postoperative day and was discharged on the 6th postoperative day.

Histopathological examination with immunohistochemical staining demonstrated aganglionosis and hypertrophic submucosal neural fibers in the biopsy from the distal narrow segment confirming the diagnosis of HD.

After confirmation of Hirschsprung's disease, Duhamel pull through procedure was done after 3 months when the general condition of the patient was optimized. Frozen section was used intraoperatively for identification of ganglionic margins. The patient started oral feeding on the 2nd postoperative day and was discharged on the 5th postoperative day. The excisional biopsy showed aganglionic distal colon and ganglionic proximal bowel. Closure of the ileostomy was 2 months later.

The patient experienced passage of loose frequent motions for the first 2 weeks after the operation which improved gradually. Currently, after 6 months from the operation, the patient is having regular bowel movement.

## 3 Discussion

HD is a common gastrointestinal disorder characterized by absence of ganglion cells in the submucosal and myenteric plexuses leading to persistent spasm and narrowing of the affected bowel segment, causing functional intestinal obstruction [7]. HD is usually diagnosed during the neonatal period, but, due to being functional disorder, the diagnosis may be delayed leading to massive dilatation of the colon which may twist if the base of the mesentery is narrow and elongated [8].

SV is a rare serious complication of HD. A recent review of the literature found only 31 cases of colonic volvulus in patients with HD [5]. Unrecognized SV is a serious life-threatening condition, so it should be considered in children with acute or recurrent abdominal pain, distension, constipation and vomiting as early recognition and management of volvulus is essential to decrease morbidity [9].

Clinical symptoms of intestinal obstruction predominate as abdominal pain, abdominal distension, constipation and vomiting. Careful interpretation of the plain abdominal radiographs recognizing a hugely dilated, twisted sigmoid loop filling the entire pelvis and abdomen with impacted stool giving a ground- glass appearance is diagnostic [10].

Management options of SV in children remain controversial but generally, non-operative reduction with enema, sigmoidoscopy or rectal tube is a possible option in clinically stable patients. However, if bowel gangrene or peritonitis is suspected, this requires immediate surgical exploration after initial resus-citation [11].

Although non-operative reduction of the SV is feasible in 30–70% it has a high recurrence rate (35%), so it should not be considered as the definitive treatment, but as temporary intervention allowing stabilization of the patient and preparation of the colon for the definitive surgery which necessitates resection of the both the aganglionic and the redundant ectatic segments and then restoring bowel continuity by anastomosis. This can be done as one stage procedure or in a staged manner [12].

In children with sigmoid volvulus, Hirschsprung's disease should be suspected, and proper evaluation is required to avoid missing the diagnosis.

**Fig. 4:**
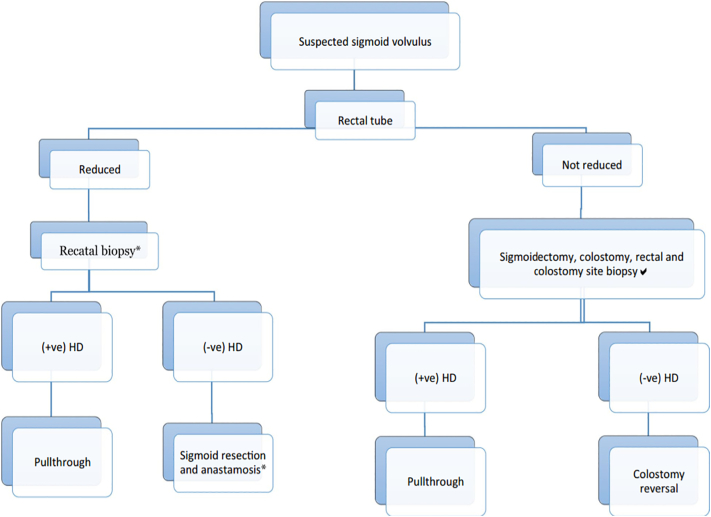
Modified Salas's et al. algorithm for the management of sigmoid volvulus in children and adolescents proposed by Abay et al. [13]. *Sigmoid resection and anastomosis should be done on semi-emergency basis as soon as rectal biopsy result arrives.

An algorithm was suggested for evaluation of these patients. Rectal tube can be used in acute sigmoid volvulus without sings of peritonitis for deflation. If deflation is successful, the tube should be kept in place for 1–2 days and rectal biopsy should be taken in the same admission. According to the biopsy result pull though or sigmoidectomy should be done urgently to protect from the high risk of early recurrence. When tube deflation is not successful or the patient develops signs of peritonitis emergency exploration is required. The options are sigmoidectomy, with either primary anastomosis or colostomy with pull through or colostomy reversal after 6–8 weeks based on the result of biopsy which is safer especially when the general condition of the patient is suboptimal [13] (Fig. 4).

In our case, there was massive colonic dilatation and ectasia till the level of the caecum with a diameter reaching 15 cm, so the decision was made for staged manner with initial diverting terminal ileostomy.

To conclude, the possibility of SV as a rare complication of HD should be considered especially in children with a history of chronic constipation and abdominal distension who present with acute colonic distension and failure to decompress despite rectal washes.

## Provenance and peer review

Not commissioned, externally peer-reviewed.

## Funding

The authors received no financial support for the research, authorship, and/or publication of this article.

## Ethical approval

This case report has been approved by local ethics committee at our institute according to the declaration of Helsinki.

## Patient consent

Written informed consent was obtained from the parents on behalf of the patient for publication of this case report and accompanying images. A copy of the written consent is available for review by the Editor-in-Chief of this journal on request.

## Author contribution

Concept – Zain; Design – Zain; Supervision – Abouheba; Materials – Zain; Data Collection And Processing – Zain; Analysis And Interpretation – Abouheba; Writer – Zain.; Critical Review – Abouheba.

## Registration of research studies

Not applicable.

## Guarantor

Mostafa Zain.

## Declaration of competing interest

The authors declared no potential conflicts of interest with respect to the research, authorship, and/or publication of this article.
